# Metformin associated lactic acidosis: a case series of 28 patients treated with sustained low-efficiency dialysis (SLED) and long-term follow-up

**DOI:** 10.1186/s12882-018-0875-8

**Published:** 2018-04-02

**Authors:** Andrea Angioi, Gianfranca Cabiddu, Maura Conti, Gianfranco Pili, Alice Atzeni, Valeria Matta, Riccardo Cao, Matteo Floris, Marco Songini, Maria Franca Mulas, Mitchell Rosner, Antonello Pani

**Affiliations:** 1 Division of Nephrology and Dialysis, Azienda Ospedaliera G. Brotzu, Piazzale Ricchi n°1, 09134 Cagliari, Italy; 2 Diabetology Unit, Azienda Ospedaliera G. Brotzu, Cagliari, Italy; 3 Division of Nephrology, University of Virginia Health System, Charlottesville, Virginia USA

**Keywords:** Lactic acidosis, Metformin, Acute kidney injury, SLED, Hemodialysis

## Abstract

**Background:**

Metformin associated lactic acidosis (MALA) is a well-known serious side effect of biguanides. However, the best treatment strategy remains a matter of debate. In the last 14 years, we observed a significant increase in hospitalizations for MALA to our Center. We report the outcomes of our clinical and therapeutic approach.

**Methods:**

This is a single-center case series. Twenty-eight patients affected with MALA and acute kidney failure admitted between January 2000 and September 2014 were included. We analyzed comorbidities, laboratory tests and clinical parameters at admission, at 36 h and at discharge. All patients were treated with sustained low-efficiency dialysis (SLED) until normalization of serum lactate (≤ 3 mmol/L), bicarbonate (between 20 and 25 mmol/L) and potassium (between 4.0 and 5.1 mmol/L).

**Results:**

The mortality rate was 21.4%, with all of the events occurring within 24 h from admission, and before or during the first hemodialysis treatment. Precipitating causes included; acute dehydration (86.4%), systemic inflammatory response syndrome (SIRS) (57.1%), sepsis (10.7%), nephrolithiasis (14.6%) and exposure to iodinated contrast (7.1%). No further episodes of lactic acidosis were described after discontinuing the drug over a mean follow-up of 27.2 months. Furthermore, while in 2010, we had a peak incidence of MALA of 76.8 cases per 100,000 patients on metformin, this rate fell after an education campaign conducted by specialists on the proper usage of metformin in patients at risk of MALA. Although the fall in incidence after the educational program was not necessarily causal, in 2014 the incidence was 32.9/100,000.

**Conclusions:**

We report an improved mortality rate in patients affected with MALA and acute kidney injury treated with SLED compared with other series published in literature. Rapid introduction of effective hemodialysis is critical in improving outcomes.

**Electronic supplementary material:**

The online version of this article (10.1186/s12882-018-0875-8) contains supplementary material, which is available to authorized users.

## Background

Metformin is a first line drug therapy both to treat type 2 diabetes mellitus as well as prevent diabetic-related complications [1–3]. It is widely considered safe and well tolerated. Mechanistically, metformin counters insulin resistance by enhancing the basal cellular uptake of glucose, and by blocking the precursors of gluconeogenesis in the liver. Clinical research demonstrated that metformin reduces postprandial glycemia by 20-30% and, as demonstrated in the United Kingdom Prospective Diabetes Study (UKPDS 34), can delay the onset of micro and macrovascular disease [2, 4–6]. Moreover, it reduces all-cause mortality (− 36%), as well as diabetes-related complications (32%) and diabetes-related deaths (− 47%) [7]. In addition, a number of pleiotropic effects have been reported such as improvement of non-alcoholic steatohepatitis [8].

Despite these benefits, some at risk populations, in particular patients with renal disease and low glomerular filtration rates (GFR) (≤ 60 ml/min) can accumulate metformin in tissues leading to lactic acidosis, which can be life threating [9]. This phenomenon is explained through in vitro and in vivo observations: metformin reduces oxidative phosphorylation, increasing serum lactate levels if drug concentrations rise excessively [10, 11]. The magnitude of metformin associated lactic acidosis (MALA) is matter of debate; in controlled studies, it is diagnosed in 4.3 per 100,000 patient-years [12]. However, several case series described a much higher incidence that likely is more reflective of the “real world” situation [13]. Metformin associated lactic acidosis (MALA) is defined as a lactic acidosis in a patient with documented regular intake of metformin as well as comorbid conditions that increase the risk of lactic acidosis such as heart failure and kidney disease [14].

Over the last fourteen years we had observed a considerable annual incidence of MALA in the setting of acute kidney injury (AKI) or acute on chronic kidney disease (AoCKD) at our Center. MALA often occurred in patients with an exacerbation of comorbid conditions. In order to better understand the etiology and outcomes of MALA, we reviewed a case series of patients who were admitted to our Center with a diagnosis of MALA and AKI. The main aim of our analysis was to evaluate the efficacy of our clinical and therapeutic approach as compared with the reported outcomes in the literature in terms of overall survival in the short (7 days) and long term (last follow-up or phone interview).

## Methods

This is a retrospective, single center, case series of MALA and AKI. The Division of Nephrology and Dialysis of the Azienda Ospedaliera G. Brotzu, Cagliari, Italy, cared for all cases. Patients included in the analysis: type 2 diabetes mellitus diagnosed at least one year earlier; metformin use as monotherapy or concomitant with other anti-diabetic medications before the development of lactic acidosis; acute kidney failure (AKI stage III) according with KDIGO Clinical Practice Guideline for Acute Kidney Injury [15]; metabolic acidosis as defined by the reduction of serum pH (≤ 7.34 [7:35-7.42]) and serum bicarbonate (≤ 22 mmol/L [22–26]); and lactic acidosis (metabolic acidosis with an increased anion gap and lactate concentration ≥ 5 mmol/L). Those patients not meeting the above listed criteria were excluded from analysis.

We identified 28 patients with a diagnosis of MALA and AKI who were admitted to our Division between January 2000 and September 2014 (Table 1). *Protocol.* Metformin was discontinued in the identified patients and treatment for diabetes was initiated with insulin and/or a low carbohydrate diet. In all cases, patients were treated with a sustained low-efficiency dialysis (SLED) using bicarbonate buffer. The goal of the treatment was metabolic normalization, in particular lactate (≤ 3 mmol/L), bicarbonate (between 20 and 25 mmol/L) and potassium (between 4.0 and 5.1 mmol/L). A metabolic panel was requested every 2 h during the treatment to prevent overcorrections and ensure that goals were being met, and every 12 h after the treatment. Of note, severe hyperkalemia was gradually corrected to ensure hemodynamic stability and avoid sudden drops of the serum concentration of potassium. In particular, if serum potassium was high (≥ 6.5 meq/l), we first corrected it with a potassium concentration of 5 in the bath and then subsequently lowered the potassium with less concentrated solutions.

**Table 1:** Descriptive statistics of selected patients at admission. M:F (male/female ratio); SD (standard deviation); CKD (chronic kidney disease)

Demographics and baseline characteristics	
Demographics	
N° of patients	28
M:F	1.5: 1
Mean age ± SD	66.7 ± 9
% of ≥ 65 years	60.7
% of ≥ 80 years	3.5
Status on admission	
N° days with symptoms ± SD before the admission	6.04 ± 5
Days of admission	10.6 ± 6.7
Systolic Pressure (mmHg)	125 ± 32.3
Diastolic Pressure (mmHg)	64.6 ± 16
Body temperature (°C)	36.9 ± 0.8
% Oliguria	71.4
Renal function	
Mean sCr (mg/dl) before the admission	1.16 ± 0.48
Mean eGFR CKD EPI (ml/min/1.73 m2) before the admission	71.9 ± 26.5
AKI stage (%)	Stage I – 0% Stage II – 3.6% Stage III – 96.4
CKD stage n (%) before the AKI	Stage I - 14 (50) Stage II - 7 (25) Stage III - 7 (25) Stage IV - 0 (0) Stage V - 0 (0)
Comorbidities	
% Heart Failure	14.2
% Iodinated contrast agent	3.5
% Moderate to severe anemia	35.7
% Vascular disease	21.4
% Non-acute pulmonary disease	7.1
% SIRS	57.1
% Sepsis	14.2
Treatment	
% Hemodialysis post-admission	100
Number of hemodialysis sessions	2.26 ± 2

Patients did not receive sodium bicarbonate i.v. before hemodialysis. Since there is little correlation between serum metformin drug levels with outcomes, metformin levels were not measured. Vital signs and laboratory studies were recorded at admission, three days after admission, and at discharge.

### Follow-up

After discharge, most of the 23 surviving patients were followed at our outpatient clinic. After a mean of 27.2 ± 24.8 months from metformin discontinuation, we asked survivors whether they experienced similar episodes in the future, about their knowledge on the drug side effects before the event and if they resumed the intake of the drug after the event.

### Educational campaign

In 2010 we observed the peak of a progressive annual rise in the incidence of MALA. This triggered us to organize several dedicated courses on MALA for specialists and general practitioners, and specific sessions in local congresses held by diabetologists, internists and nephrologists.

Laboratory parameters are expressed as mean and standard deviation (SD) for normally distributed variables and median and interquartile range for non-parametric data. The evolution of lab exams at admission and after 3 days was reported and differences were assessed with the paired t-test. Statistical analysis was carried out using SPSS 22 (IBM Corporation, USA).

We defined systemic inflammatory response syndrome (SIRS) and sepsis as recommended by ACCP/SCCM (2012) [16].

## Results

We identified 28 patients diagnosed with MALA from our hospital records. There was a prevalence of males (M:F 1.5:1), and the mean age was 66.7 ± 9.5 yrs. Clinical and laboratory findings at admission were diagnostic for AKI stage 2 in 3.6% and stage 3 in 96.4% of patients (Table 1). However, after the first 24 h of observation, all patients met criteria for AKI stage III. Notably, 50% of MALA cases were observed in subjects with previously normal kidney function, and in 25% of cases, MALA developed in subjects with previously diagnosed mild to moderate chronic kidney disease (sCr 1.16 ± 0.48 mg/dl; eGFR CKD-EPI 71.9 ± 26.5 ml/min/1.73 m2) (Table 2). Comorbid conditions included: hypertension (71.4%), neoplastic disease (17.9%), chronic obstructive pulmonary disease (COPD) (7.1%) and heart failure (14.3%) (Additional file 1: Table S1). We did not identify any combination of comorbidities associated with an increased risk for mortality. Our cohort was treated as follows: a mean of 6.9 ± 1.8 h of treatment were provided with low blood fluxes (QB from 80 to 140 ml/min) due to cardiovascular instability and mono-lumen catheters that we use for an urgent dialysis; dialyzers were based on multi-branded polysulfone membranes.

**Table 2:** Laboratory parameters at time 0 (admission) and at 36 h, intended as means and standard deviation (SD); EB (excess of bases), pCO_2_ (CO_2_ partial blood pressure)

Laboratory parameters at time 0 (admission) and at 36 h, intended as means and standard deviation (SD)			
Variable (mean ± SD)	*Time 0*	*Time 1 (36 h)*	*Sig.*
Serum Creatinine (mg/dl)	8.1 ± 3.1	5.3 ± 2.8	*p < 0.05*
Blood Urea Nitrogen (mg/dl)	96.8 ± 47.4	54.6 ± 27.4	*p < 0.05*
Hemoglobin (g/dl)	10.7 ± 1.6	11.2 ± 1.8	*p NS*
Potassium (mEq/L)	5.93 ± 1.4	4.03 ± 0.8	*p < 0.05*
Glycemia (mg/dl)	178.8 ± 123	160.1 ± 24	*p NS*
C-reactive protein (mg/dl)	8.6 ± 8.9	4.3 ± 2.3	*p < 0.05*
pH, serum	7.01 ± 0.22	7.35 ± 0.7	*p < 0.05*
HCO_3_ -(mmol/L)	7.72 ± 4.4	22.7 ± 6.4	*p < 0.05*
EB (mmol/L)	−19.3 ± 11.7	−1.92 ± 5.6	*p < 0.05*
Lactate (mmol/L)	13.7 ± 6	2.6 ± 2.6	*p < 0.05*
Anion gap (mmol/L)	36.2 ± 8.2	13.2 ± 4.1	*p < 0.05*
VpCO_2_ (mEq/L)	25.7 ± 9.5	39.1 ± 4.4	*p < 0.05*
VpO_2_ (mEq/L)	65.4 ± 10.5	42.4 ± 9.9	*p < 0.05*

A limited number of causes in this patient cohort that could worsen kidney function were identified. These included pre-renal etiologies, volume depletion (85.7%) secondary to nausea and vomiting (53.6% and 57.1%, respectively). Nephrolithiasis caused post-renal acute renal failure in 14.3% of patients. Systemic inflammatory response syndrome (SIRS) was frequently diagnosed (57.1%), and 10.7% of hospitalized patients had criteria diagnostic of sepsis (*E. coli* was the most frequent pathogen (2/4 cases)). Two patients developed AKI after administration of iodinated contrast medium, developing MALA because metformin had not been discontinued. Mild (10-12 g/dl) to moderate (8-9.9 g/dl) anemia often concurred in this condition (37.5%).

**Fig. 1:**
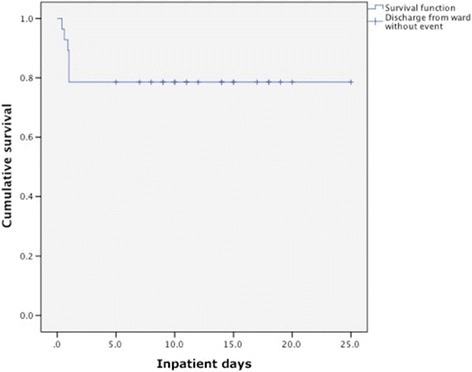
Kaplan-Meier survival curve of admitted-discharged patients affected with MALA (admission, time 0 – 25 days). Discharged patients are marked as censored. During the follow-up (mean 27.2 months), none of the surviving patients experienced new episodes of lactic acidosis after discontinuing the drug. Two patients died at follow-up for reasons other than lactic acidosis

Elevated levels of serum creatinine (mean: 8.1 ± 3.1 mg/dL) and blood urea nitrogen (mean: 96.8 ± 47.4 mg/dL), as well as hyperkalemia (mean: 5.93 ± 1.4 mEq/L), low pH values (median: 7.01 ± 0.22) and elevated blood lactate values (median: 13.7 ± 6 mEq/L) characterized patients at admission. After their first emergency hemodialysis, 21.4% of patients died. This occurred within the first 24 h of admission (Fig. 1). Mean hospitalization length was 10.6 ± 6.7 days, and a mean of only 2.26 ± 2 hemodialytic sessions (HD) were performed. Kidney function rapidly recovered in the surviving patients, and in some cases within 36 h. Metabolic parameters also normalized significantly within the same time frame (pH [7.36 ± 0.072], HCO3- [22.71 ± 6.44 mmol/L], lactate levels [2.6 ± 2.65 mmol/L]). Patients who were oliguric at admission recovered urine output within 36 h. At discharge, metabolic parameters were still within normal range, but 14.8% of patients did not recover to their previous values of renal function. We did not observe any MILA, unlike occasionally reported in the literature [17, 18].

These subjects were then followed-up as outpatients for a mean of 27.2 months in order to assess changes in kidney function and to document whether lactic acidosis reappeared despite metformin discontinuation. Telephone interviews allowed us to collect data from the other patients cared for in other facilities. A brief interview with the patients and their relatives revealed that: 1) none of them knew how to recognize either the initial symptoms of MALA or the signs and risks of sudden decline in kidney function. Above all, none of the patients knew when to discontinue metformin if they developed an illness and therefore they all continued taking the drug until admission to hospital; 2) after discharge, 2 of 23 patients died from causes other than lactic acidosis; 3) no new episodes of lactic acidosis were reported; 4) the onset of symptoms that led to a diagnosis of MALA started a mean of 6 days before admission.

Considering that our operative area accounts for 552.303 inhabitants and that 1.55 individuals out of every 100 have a prescription for metformin, we estimated that in our district there are about 8560 patients taking metformin. Thus, in 2010, we had a peak incidence of MALA of 76.8 cases per 100,000 patients on metformin, that fell after an education campaign conducted by specialists on its usage in patients at risk of MALA (Additional file 2: Figure S1). Although the fall in incidence after the educational program is not necessarily causal, in 2014 the incidence was 32.9/100,000, while prescription rate remained almost stable in this period.

## Discussion

Currently, type 2 diabetes is progressing at alarming rates in Europe, US and Italy [19, 20]. Although there are no official data regarding the incidence of T2D in Sardinia, some regional reports show a growing tendency, estimated at about 5000 new cases every year. The number of affected subjects is estimated around 94,000 [20]. In Italy, antidiabetic drug use increased by 37% between 2000 and 2009 [3], especially oral anti-diabetics (+ 22%). Prescriptions for metformin as the initial treatment for incident cases of type 2 diabetes increased exponentially, going from 8.3% in 1998 to 54.3% in 2010 [21]. The result of an increase in the number of prescriptions is, of course, an increase in the number of reported adverse events [22]. The most serious side effect of metformin is lactic acidosis, a form of severe metabolic acidosis with a concomitant increase in serum concentrations of lactate ≥ 5 mmol/L. This is the result of blockade of pyruvate flow through its metabolic pathway induced by high levels of metformin. Pyruvate is the product of glucose metabolism through glycolysis and subsequently enters into the mitochondrial Krebs Cycle and oxidative phosphorylation. This blockade leads to a significant decrease in ATP generation and an increase in protons, resulting in a consequent reduction of pH in the cytoplasmic fluid due to continuous ATP hydrolysis [21]. This ultimately leads to a block in pyruvate metabolism and the excess pyruvate that cannot be managed by the mitochondria is transformed into lactate, especially within the liver, intestine and, to a lesser extent, in other tissues [23, 24]. It is well known that MALA often occurs as the consequence of an abrupt increase in the concentration of metformin in tissues and serum, often following a rapid decline in kidney function and decrease in metformin excretion [25].

Although rare, MALA generated a great deal of concern that delayed the introduction of metformin into the US market until May, 1995 [26]. This was only after extended studies and meta-analyses determined that the condition was rare especially in those with normal kidney function [22, 27]. A Cochrane meta-analysis carried out by Salpeter et al. included reports published between January, 1959 and October, 2009, and involved 70,490 patients who had taken metformin for at least 1 month [12]. The results showed an annual MALA incidence of 4.3 cases per 100,000 patient-years in the metformin group and 5.4 per 100,000 patient-years in the non-metformin group, resulting in a paradoxical protective effect of metformin against lactic acidosis due to other causes. However, the work by Salpeter actually estimated the incidence of MILA, a lactic acidosis generated by a metformin poisoning *alone* and known to be quite uncommon, instead of MALA. In fact, it differs significantly from the “real world” experience, where several comorbidities able to generate lactic acidosis may easily coexist in our patients.

Of note, many cases of MALA may have been prevented before “the point of no return” by discontinuing the drug at the onset of suspicious symptoms or when patients developed conditions such as volume depletion which would reduce GFR and increase metformin levels. So far in 2014, the incidence of the phenomenon is half what it was in 2010, but still high, as reported in other recent papers [28]. Moreover, the National Pharmacovigilance Network, a system for data collection and management of suspected adverse reactions of the Italian Medicines Agency, analyzed the cases of MALA reported only 59 new cases of MALA in the 10 year period November 2001 to October 2011, suggesting elevate rates of missed reports [29].

Our patients were affected by comorbidities that may themselves generate lactic acidosis independently of metformin use. However, arguing against this is that after discontinuing the drug, there were no further events. In fact, MALA is currently the main etiology of lactic acidosis among the patients in our ward. Mortality was relatively low in our cohort (21.4%) and all deaths occurred within 24 h of admission. A review of electrocardiographic data in these deaths revealed ventricular tachycardia first, then followed by ventricular fibrillation. The 24-h critical limit for mortality has also been reported by other authors in the literature [27]. Mortality was below 45% in a meta-analysis by Lalau et al., but higher compared to our data [30]. Another report from Seidowsky et al showed even a higher mortality incidence of 48% [31].

The death events occurred during the first hemodialytic treatment (after a mean of 3:15 h of treatment, around 5 h since admission) or soon after (two patients died 24 h since admission and one patient after 22 h). Patients had very similar clinical presentations, commonly with moderate to severe dehydration associated with vomiting, diarrhea and oliguria. Upon admission to our Division, blood gases often showed high levels of venous pO2, most likely because of hyperventilation secondary to severe acidosis and the exclusion of the Krebs cycle from cellular metabolism due to metformin intoxication, in agreement with Protti et al [32].

We believe that the improved mortality seen in our cohort is due to aggressive use of SLED along with more gradual correction of metabolic derangements. Interestingly, recent guidelines had been published by the EXTRIP workgroup, that are much more conservative compared with our approach. In fact, hemodialysis is indicated if any of the following conditions (lactate concentration greater than 20 mmol/L; pH less than or equal to 7.0; shock; Failure of standard supportive measures; decreased level of consciousness) were present. Instead, we preferred to be more aggressive with the use of dialysis, prescribing SLED in every patient taking metformin with lactic acidosis (metabolic acidosis with an increased anion gap and lactate concentration ≥ 5 mmol/L) and AKI stage III.

The renal replacement therapy utilized in our protocol was based on a bicarbonate buffer and intermittent SLED to ensure adequate clearance of metformin. After the first SLED and volume correction, a second session was often needed (mean: 2.26 ± 2 treatments) to achieve lactate ≤ 5 mmol/L. This is in agreement with the kinetics of the drug, resulting in complete removal in about 48 h [33]. Very slow correction of potassium was critical to avoid polarization abnormalities in conduction tissues, especially in subjects whose K^+^ ≥ 7 mEq/L. The use of these strategies led to an improvement in renal function within 3 days of admission, thus allowing patients to be weaned from replacement therapy. Prolonged hospitalization (mean: 10.6 ± 6.7 days) was often necessary to complete the diagnostic and therapeutic plan and to stabilize all clinical parameters, including control of glycemia with the new insulin regimens. Unfortunately, 14.8% of patients did not regain their previous eGFR values. No other deaths due to lactic acidosis were reported a mean of 27.2 months after discontinuing metformin, and no new episodes of lactic acidosis were observed. This is an indirect sign of the key role of metformin in the genesis of MALA.

Our study has several limitations. First, given the small number of patients in our study, we did not find any correlation between death and clinical or laboratory variables. Theoretically, comorbidities could influence this outcome, but neither respiratory, cardiac, nor renal disease had a clear impact on outcome in this case series. This suggests that MALA may be fatal independently from comorbidities. Overall, there is very little information available to predict the evolution of this condition [21]. Second, we did not provide any control group to demonstrate a significant improvement of survival in MALA compared to other conditions characterized by lactic acidosis.

## Conclusions

In conclusion, we report a high incidence (in 2014, 32.9/100,000 patient) but a relatively low mortality rate (21.4%) of MALA. MALA tended to be precipited by AKI mainly due to volume depletion. Importantly, patients had little insight into how to manage their metformin dosing as they continued their daily intake of the drug until toxic levels were reached despite having conditions that increased risk such as nausea and vomiting. SLED hemodialysis and a very slow correction of the metabolic profile was effective and led to satisfactory outcomes in patients with MALA.

## Additional files

Additional file 1: **Table S1.** Comorbidities and precipitating factors associated with lactic acidosis (MALA). Death events are marked red. (PPTX 47 kb)

Additional file 2: **Figure S1.** Incidence of MALA observed to our Division between January 2000 and September 2014. It is indicated the extensive educational campaign done by our Division for internal medicine and metabolism specialists. (PPTX 105 kb)

## Abbreviations

AKI: Acute kidney injury
AoCKD: Acute on chronic kidney disease
COPD: Chronic obstructive pulmonary disease
eGFR: Estimated glomerular filtration rate
HD: Hemodialysis
KDIGO: Kidney Disease: Improving Global Outcomes
MALA: Metformin associated lactic acidosis
MILA: Metformin induced lactic acidosis
SIRS: Systemic inflammatory response syndrome
SLED: Sustained low efficiency dialysis
T2D: Type 2 diabetes
