# Supplementary material for: Metformin associated lactic acidosis: a case series of 28 patients treated with sustained low-efficiency dialysis (SLED) and long-term follow-up
Source: BMC Nephrol. 2018 Apr 2;19:77. doi: 10.1186/s12882-018-0875-8 (PMC5879547; doi:10.1186/s12882-018-0875-8)
Supplement: Supplementary file 1 — Table S1. Comorbidities and precipitating factors associated with lactic acidosis (MALA). Death events are marked red. (PPTX 47 kb) [file 12882_2018_875_MOESM1_ESM.pptx]

## Slide 1
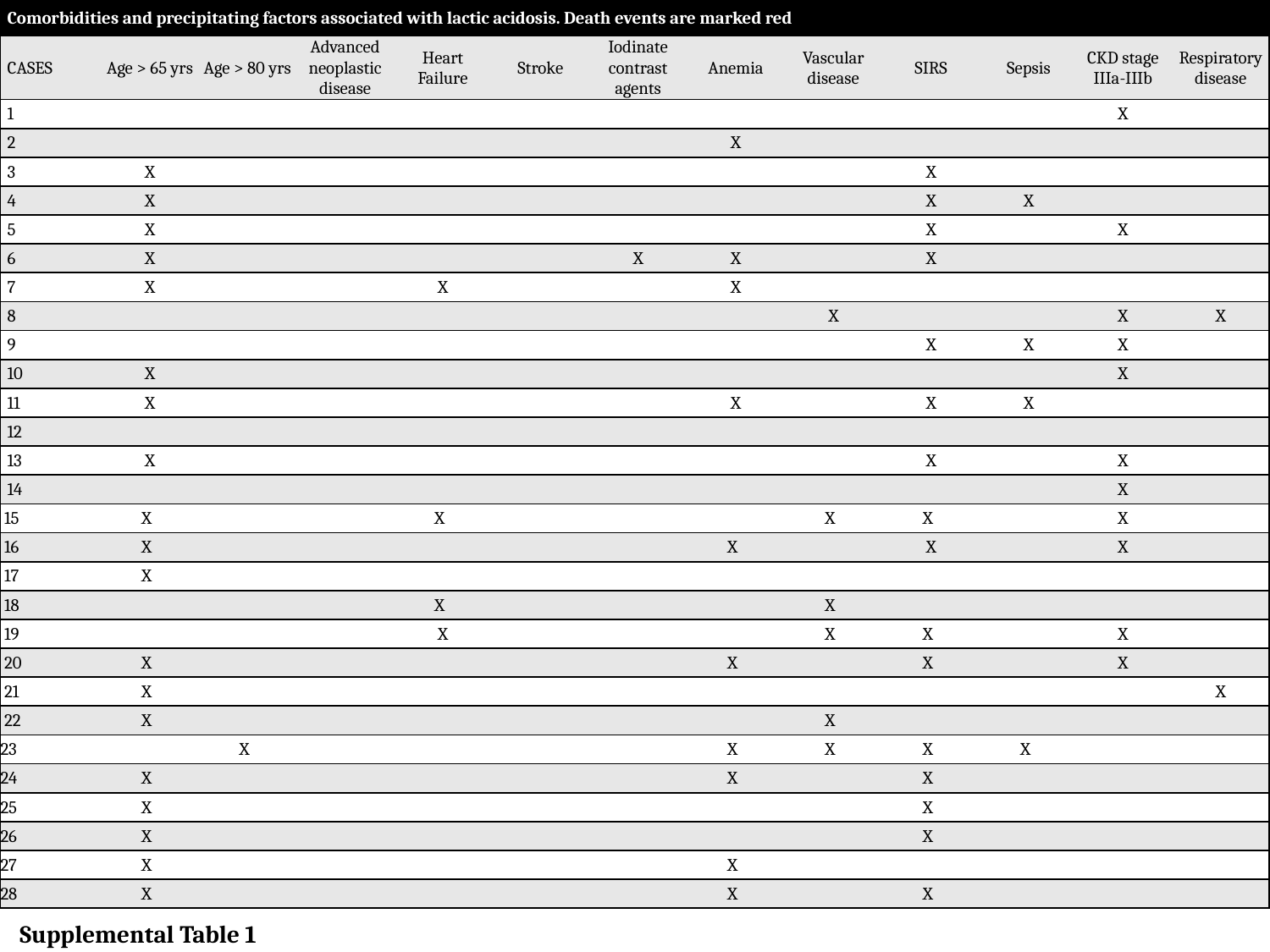

| Comorbidities and precipitating factors associated with lactic acidosis. Death events are marked red | | | | | | | | | | | | |
| --- | --- | --- | --- | --- | --- | --- | --- | --- | --- | --- | --- | --- |
| CASES | Age > 65 yrs | Age > 80 yrs | Advanced neoplastic disease | Heart Failure | Stroke | Iodinate contrast agents | Anemia | Vascular disease | SIRS | Sepsis | CKD stage IIIa-IIIb | Respiratory disease |
| 1 | | | | | | | | | | | X | |
| 2 | | | | | | | X | | | | | |
| 3 | X | | | | | | | | X | | | |
| 4 | X | | | | | | | | X | X | | |
| 5 | X | | | | | | | | X | | X | |
| 6 | X | | | | | X | X | | X | | | |
| 7 | X | | | X | | | X | | | | | |
| 8 | | | | | | | | X | | | X | X |
| 9 | | | | | | | | | X | X | X | |
| 10 | X | | | | | | | | | | X | |
| 11 | X | | | | | | X | | X | X | | |
| 12 | | | | | | | | | | | | |
| 13 | X | | | | | | | | X | | X | |
| 14 | | | | | | | | | | | X | |
| 15 | X | | | X | | | | X | X | | X | |
| 16 | X | | | | | | X | | X | | X | |
| 17 | X | | | | | | | | | | | |
| 18 | | | | X | | | | X | | | | |
| 19 | | | | X | | | | X | X | | X | |
| 20 | X | | | | | | X | | X | | X | |
| 21 | X | | | | | | | | | | | X |
| 22 | X | | | | | | | X | | | | |
| 23 | | X | | | | | X | X | X | X | | |
| 24 | X | | | | | | X | | X | | | |
| 25 | X | | | | | | | | X | | | |
| 26 | X | | | | | | | | X | | | |
| 27 | X | | | | | | X | | | | | |
| 28 | X | | | | | | X | | X | | | |
Supplemental Table 1
