# Supplementary figures and images for: Metformin associated lactic acidosis: a case series of 28 patients treated with sustained low-efficiency dialysis (SLED) and long-term follow-up
Source: BMC Nephrol. 2018 Apr 2;19:77. doi: 10.1186/s12882-018-0875-8 (PMC5879547; doi:10.1186/s12882-018-0875-8)

## Slide 1
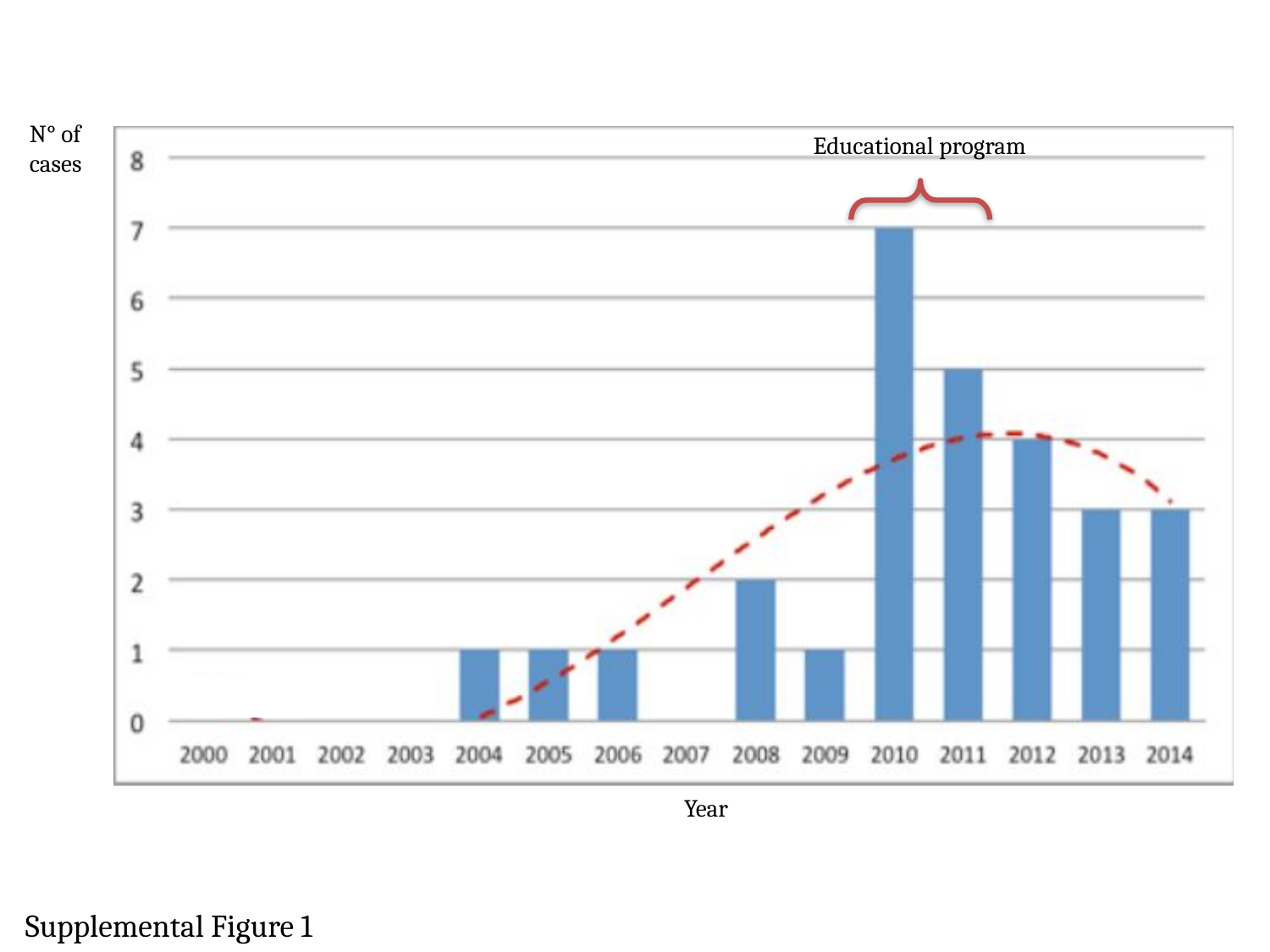

N° of
cases
Educational program
Year
Supplemental Figure 1

Supplement: Supplementary file 2 — Figure S1. Incidence of MALA observed to our Division between January 2000 and September 2014. It is indicated the extensive educational campaign done by our Division for internal medicine and metabolism specialists. (PPTX 105 kb) [file 12882_2018_875_MOESM2_ESM.pptx]
